# Five-Membered Rings Create Off-Zero Modes in Nanographene

**DOI:** 10.1021/acsnano.3c06006

**Published:** 2023-12-05

**Authors:** Peter H. Jacobse, Michael C. Daugherty, Kristia̅ns Čerņevičs, Ziyi Wang, Ryan D. McCurdy, Oleg V. Yazyev, Felix R. Fischer, Michael F. Crommie

**Affiliations:** † Department of Physics, University of California, Berkeley, California 94720, United States; ‡ Department of Chemistry, University of California, Berkeley, California 94720, United States; § Institute of Physics, Ecole Polytechnique Fédérale de Lausanne (EPFL), 1015 Lausanne, Switzerland; ∥ Materials Sciences Division, Lawrence Berkeley National Laboratory, Berkeley, California 94720, United States; ⊥ Kavli Energy NanoSciences Institute at the University of California, Berkeley and the Lawrence Berkeley National Laboratory, Berkeley, California 94720, United States; # Bakar Institute of Digital Materials for the Planet, Division of Computing, Data Science, and Society, University of California, Berkeley, California 94720, United States

**Keywords:** nanographenes, five-membered rings, electronic structure, magnetic ground state, open shell, zero-modes, scanning tunneling microscopy

## Abstract

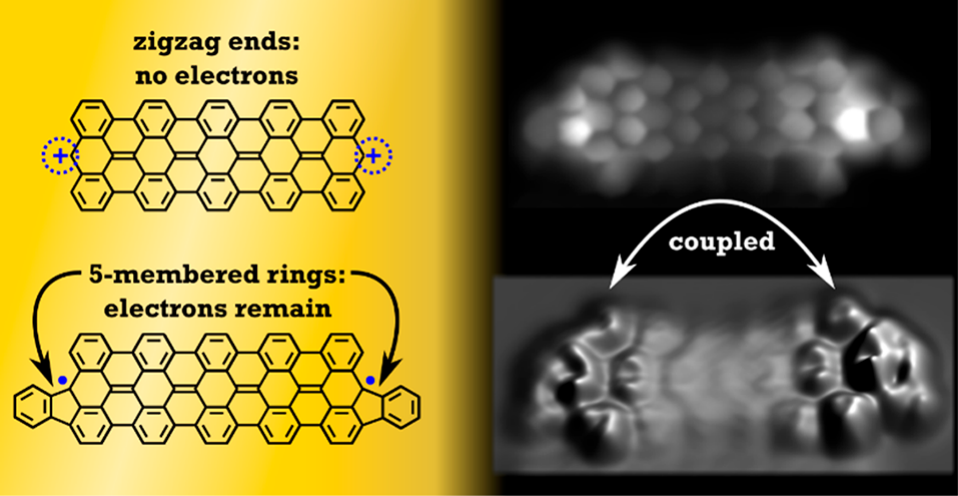

The low-energy electronic structure of nanographenes can be tuned through zero-energy π-electron states, typically referred to as zero-modes. Customizable electronic and magnetic structures have been engineered by coupling zero-modes through exchange and hybridization interactions. Manipulation of the *energy* of such states, however, has not yet received significant attention. We find that attaching a five-membered ring to a zigzag edge hosting a zero-mode perturbs the energy of that mode and turns it into an *off-zero* mode: a localized state with a distinctive electron-accepting character. Whereas the end states of typical 7-atom-wide armchair graphene nanoribbons (7-AGNRs) lose their electrons when physisorbed on Au(111) (due to its high work function), converting them into off-zero modes by introducing cyclopentadienyl five-membered rings allows them to retain their single-electron occupation. This approach enables the magnetic properties of 7-AGNR end states to be explored using scanning tunneling microscopy (STM) on a gold substrate. We find a gradual decrease of the magnetic coupling between off-zero mode end states as a function of GNR length, and evolution from a more closed-shell to a more open-shell ground state.

Synthetic capabilities to fabricate atomically precise nanographenes (NGs) have increased tremendously over the past decade, resulting in an ability to explore and tailor their intrinsic electronic properties.^1−4^ NGs are quantum-confined substructures of the graphene lattice and typically exhibit a sizable energy gap unlike semimetallic extended 2D graphene.^5−8^ In recent years local sublattice imbalances,^9−14^ the substitution of heteroatoms,^15−21^ and the incorporation of four-, five-, seven-, and eight-membered rings^22−28^ have been used to engineer low-energy electronic states in NGs. Such low-energy states hybridize and correlate, ultimately leading to the emergence of properties including magnetism and metallicity.^29−34^ Engineering localized low-energy states thus provides a tool for bypassing the natural tendency of aromatic structures to exhibit a gapped, closed-shell electronic structure and creating emergent low-energy electronic behavior instead. These bottom-up nanostructures have potential for future applications in carbon-based nanoelectronics and quantum information processing.^35−41^

A popular method for engineering low-energy states in NGs is the design of sublattice imbalance in the honeycomb lattice, which generates zero-energy states (or zero-modes) per Lieb’s theorem.^42−44^ But even when the number of atoms on the A and B sublattices in a NG are equal, zero-modes can still be triggered by local sublattice imbalances as per Ovchinnikov’s rule or due to topological frustration.^45−48^ Zero-modes also arise more generally whenever an open-shell (or spin-split) ground state is lower in energy than its closed-shell counterpart, and this can be caused by the open-shell resonance structure featuring greater aromatic stabilization.^32,48^ 7-atom wide armchair-type graphene nanoribbons (7-AGNRs) demonstrate this behavior by featuring an open-shell biradical ground state with two characteristic end-localized states.^49,50^ In short 7-AGNRs, however, end states may hybridize and thus introduce closed-shell character to the ground state. Anthracene and bisanthene, for example, exhibit primarily closed-shell ground states.^51^ The zero-energy nature of zero-modes typically implies that these localized states have magnetic moments, since at charge neutrality they are usually singly occupied.^49^ Hence, zero-modes in close proximity can couple through exchange interactions to impart magnetic behavior to NGs. For 7-AGNRs a length-dependent exchange-coupling strength *J* between spins has been predicted on top of the transition from a closed-shell to an open-shell ground state.^52,53^ Unfortunately the magnetic characteristics of 7-AGNRs cannot be studied on a gold surface (typically used for the synthesis of 7-AGNRs) because of p-doping by the surface which is known to extract electrons from the end states.^50^ In the absence of electron occupation the end states only interact through hybridization, which has recently been shown between empty 7-AGNR end states in teranthene (three repeating anthracenes) and hexanthene (six repeating anthracenes) on Au(111).^54^

Here we describe a strategy to create localized states, hereafter termed off-zero modes, that are energetically offset compared to their zero-mode counterparts. Off-zero modes can be thought of as basis states for generating designer quantum structures in NGs (see Supplementary Discussion 1). Our strategy leverages the intrinsic stabilizing effect imparted by electron-withdrawing cyclopentadienyl groups: the five-membered rings in fluorenyl radicals. Fusion of cyclopentadienyl groups to the zigzag ends of 7-AGNRs prevents electron transfer from the 7-AGNR to the underlying gold that would otherwise leave these states unoccupied.^50^ We exploit this phenomenon to perform a systematic scanning tunneling microscopy (STM) and spectroscopy (STS) study of the magnetic coupling between off-zero modes localized at the ends of 7-AGNR oligomers as a function of their separation while supported by a Au(111) substrate.^53−57^ We identify singlet-to-triplet spin-flip excitations for 7-AGNRs (oligoanthenes) having three and four repeating anthracene units and a Kondo effect for five and six repeating units, reflecting a monotonically decreasing exchange-coupling interaction with increasing separation. Differential conductance maps suggest significant closed-shell character for shorter 7-AGNRs. Our study thus reveals an evolution from a more closed-shell to a more open-shell ground state in 7-AGNR segments with increasing length.

## Results/Discussion

### Five-Membered-Ring-Induced Off-Zero Modes

**Figure 1:**
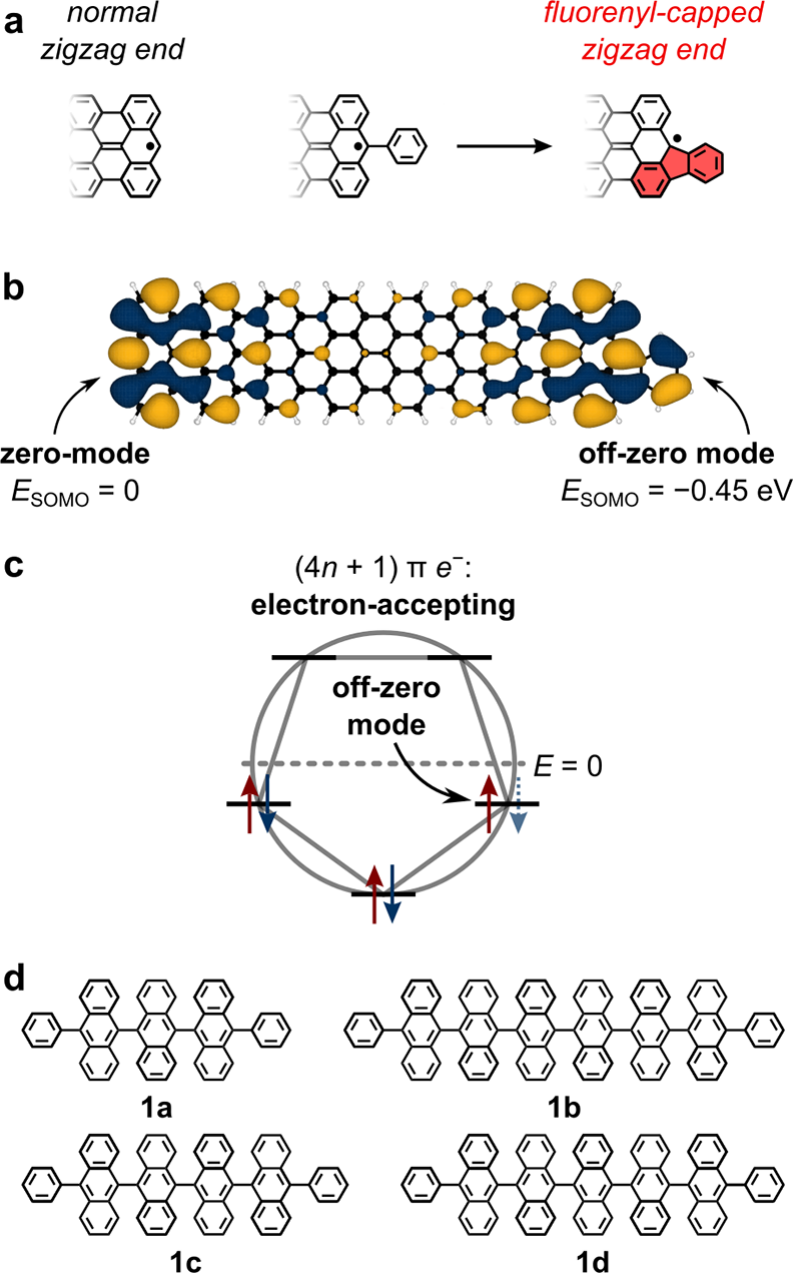
Off-zero modes. (a) Normal 7-AGNR zigzag end with a radical end state (left) and a fluorenyl-capped zigzag end (right) obtained by fusion of a pendant phenyl group (center). The fluorenyl moiety perturbs the state associated with the radical. (b) Result of a DFT calculation on a 7-AGNR with *n* = 7 repeating anthracene units that is capped with a fluorenyl on the right end only. The SOMOs and their respective energies are shown. (c) Frost circle for cyclopentadienyl. The frontier states lie below *E* = 0. (d) Chemical structure of precursors **1a**−**d**.

The design of our off-zero mode structures is guided by density functional theory (DFT) simulations of a finite 7-AGNR featuring one pristine zigzag end, while the second end is capped by a fluorenyl group (Figure 1a,b). Our calculations predict an open-shell ground state for this GNR, with a singly occupied molecular orbital (SOMO) localized on the capped end 0.45 eV lower in energy than the SOMO on the pristine zigzag end (Figure 1b). The electronic stabilization gained by introducing the cyclopentadienyl ring thus converts the end-state *zero-mode* to a lower-energy *off-zero mode*. This behavior can be rationalized using the Frost circle heuristic wherein a planar, monocyclic, and conjugated ring of five C atoms exhibits an electron-accepting state at *E* < 0 (Figure 1c).^58^ The DFT-calculated density of states (DOS) of a semi-infinite 7-AGNR terminated by either a pristine zigzag end or a cyclopentadienyl ring shows similar behavior (Supplementary Discussion 2 and Figure S1). Interestingly, the Frost circle model also implies that the introduction of a 7-membered ring, i.e., a cycloheptatrienyl group, would give rise to the opposite effect: off-zero modes with *E* > 0 and thus electron-donating character.

### Synthesis of Fluorenyl-Capped Oligoanthenes

**Scheme 1:**
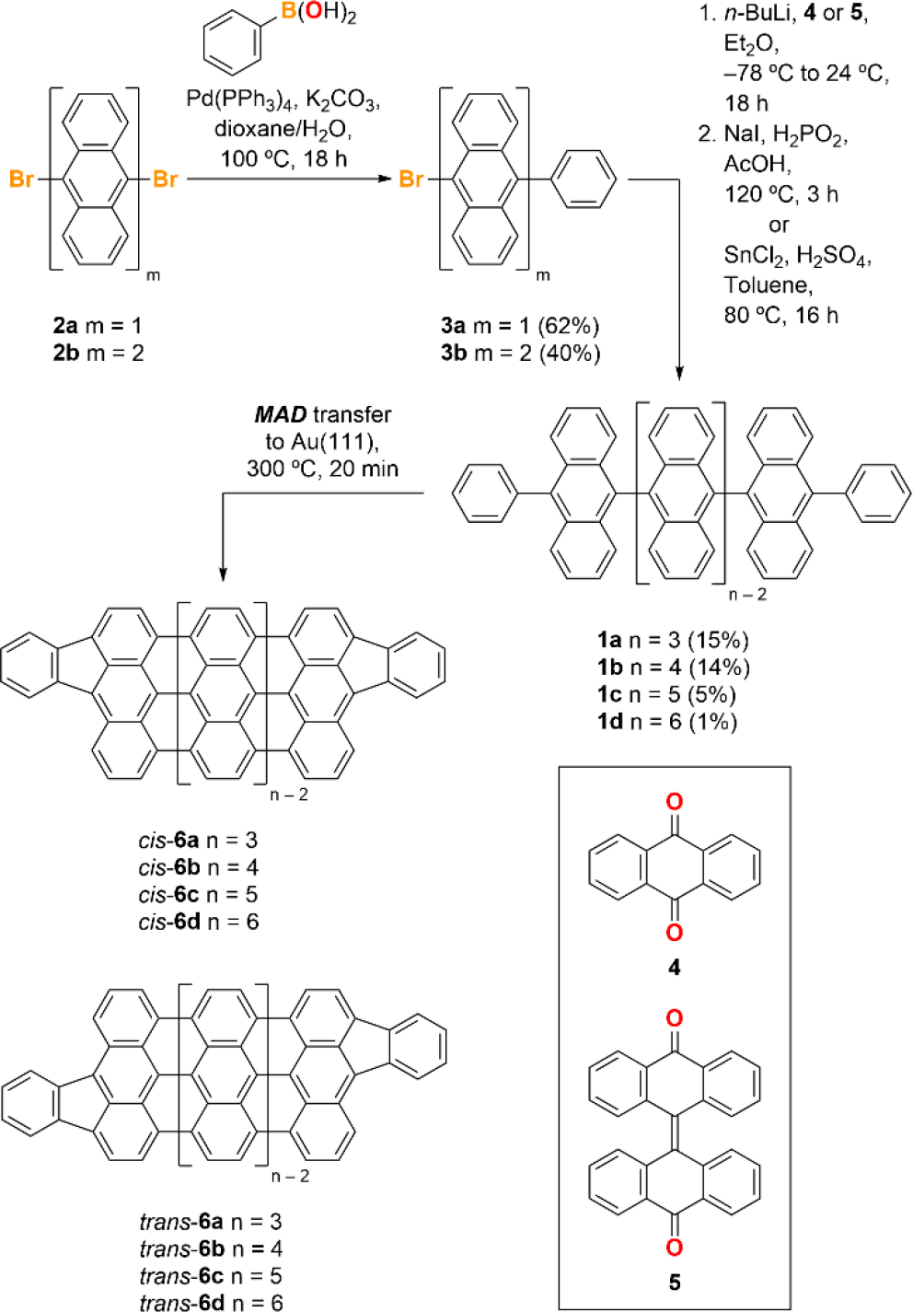
Synthesis of Oligoanthenes **6a**–**d**

To introduce fluorenyl end-groups to 7-AGNRs, we took advantage of the known propensity of pendant phenyl groups bound to zigzag NG edges to form cyclopentadienyl rings upon cyclodehydrogenation (CDH) (Figure 1a).^59−62^ We designed four different phenyl-terminated oligoanthene precursors: diphenylteranthryl (**1a**), diphenylquateranthryl (**1b**), diphenylquinqueanthryl (**1c**), and diphenylsexianthryl (**1d**) (Figure 1d and Scheme 1). The synthesis of all four molecular precursors (**1a**–**d**) started with the Suzuki–Miyaura cross-coupling of dibromoanthracene **2a** or dibromobianthracene **2b** with one equivalent of phenylboronic acid to give the corresponding phenyl-capped monobromides **3a**,**b**. Lithiation of **3a**,**b**, nucleophilic addition to anthraquinone (**4**) or bianthrone (**5**), and subsequent dehydroxylation affords oligoanthrylene products **1a**–**d**. Details of the chemical synthesis and characterization are provided in the Supporting Information (Scheme S1, S2 and Figures S2–S3).

**Figure 2:**
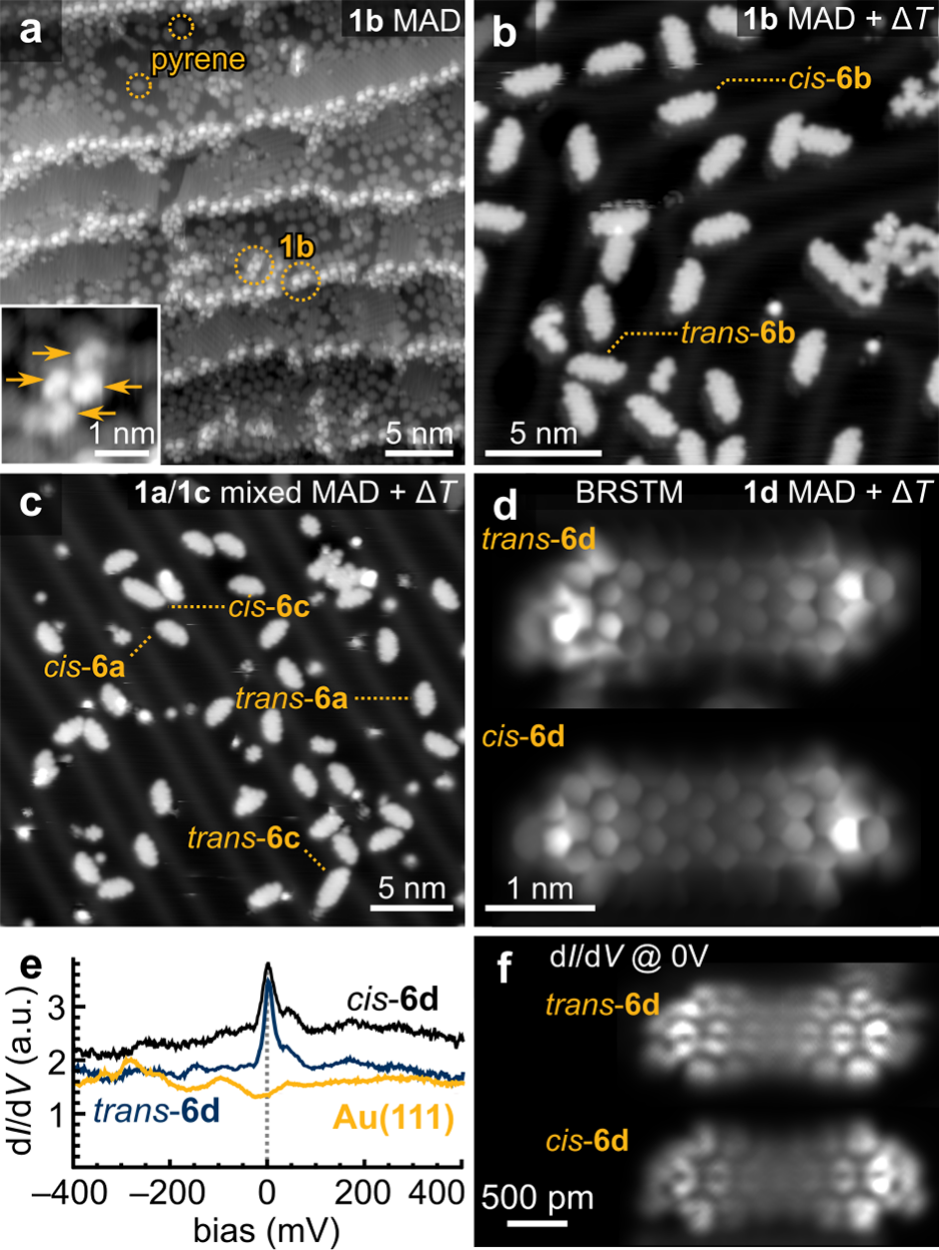
Synthesis of fluorenyl-capped oligoanthenes. (a) STM topographic image (*V* = −1800 mV, *I* = 50 pA) of a sample of **1b** on Au(111) after MAD transfer. The inset shows a single molecule of **1b**. (b) STM topographic image (*V* = −300 mV, *I* = 50 pA) of a sample of **6b** on Au(111) obtained after MAD transfer of **1b** followed by heating to *T* = 300 °C for 20 min. (c) STM topographic image (*V* = −1600 mV, *I* = 50 pA) of a mixed sample of **6a** and **6c** on Au(111) obtained after MAD transfer of a mixture of **1a** and **1c** followed by heating to *T* = 300 °C for 20 min. (d) BRSTM images (*V* = −300 mV, *V*_ac_ = 100 mV) of *cis*-**6d** and *trans*-**6d** obtained after MAD transfer of **1d** followed by heating to *T* = 300 °C for 20 min. (e) d*I*/d*V* spectra (*V*_ac_ = 2 mV) recorded at the end of *trans*-**6d** (black) and *cis*-**6d** (blue). (f) Differential conductance maps (*V* = 0, *V*_ac_ = 10 mV, constant height) of the zero-bias resonance on *trans*-**6d** and *cis*-**6d**. All STM measurements were recorded at *T* = 4.5 K.

The extremely low vapor pressure of oligoanthrylenes precludes thermal sublimation, and so we relied on a matrix-assisted direct (MAD) transfer technique to deposit these molecules onto Au(111) substrates suitable for on-surface CDH and STM characterization.^63,64^Figure 2a shows an STM image of a Au(111) surface after MAD transfer of **1b** in a pyrene matrix, followed by gradual heating to *T* = 200 °C for *t* = 1 h to remove the bulk of the pyrene. Molecules of **1b** are recognizable by the presence of four lobes that result from the dihedral angle between neighboring anthracenes (inset of Figure 2a) and are preferentially localized along the Au(111) step edges. Figure 2b shows the sample after further heating to the CDH temperature *T*_CDH_ = 300 °C for *t* = 20 min. The molecules have fully cyclized, yielding the planar quateranthene **6b**, while almost all the residual pyrene has sublimed from the surface. The fin-shaped ends observed for **6b** suggest that the phenyl rings have fused with the zigzag end forming the expected terminal fluorenyl groups (Figure 2b). Since the phenyl rings can fuse with either *peri* position on the terminal anthracene unit, the surface shows a mixture of *C*_2*v*_ and *C*_2*h*_ symmetric molecules representing the *cis* and *trans* configurations, respectively. The three other capped oligoanthenes (**6a**, **6c**, and **6d**) were prepared in a similar fashion. Figure 2c shows a topographic image of a sample containing **6a** and **6c** on Au(111), generated from a mixture of **1a** and **1c** in pyrene. Figure 2d shows bond-resolved STM (BRSTM) images of the two configurational isomers of **6d**, corroborating the structure of the sexianthene molecular core terminated on either end by fluorenyl groups.^65,66^ The five-membered rings of the fluorenyl groups exhibit high brightness in the BRSTM image, consistent with the localization of off-zero modes at the ends of the GNRs.

Confirmation of the electron occupation of the off-zero modes was obtained by scanning tunneling spectroscopy (STS). The longest oligoanthene examined here, *cis*/*trans*-**6d**, provides a useful platform to gauge electron occupation since the hybridization between the end states is expected to be negligibly small.^56^ STS recorded on the ends of **6d** reveals a narrow zero-bias peak, indicative of a Kondo resonance (Figure 2e). Here the independent magnetic moments of the end-state electrons are screened by itinerant electrons of the gold.^29^ The SOMOs associated with the end state can be imaged by spatially mapping the differential conductance at zero bias (Figure 2f). The observation of a Kondo resonance contrasts with STS of pristine 7-AGNR end states on Au(111) where the zero-modes are observed at positive bias and the Kondo peak is absent, consistent with these states being vacant due to p-doping from the Au(111) substrate.^50^

### Experimental Length Dependence of the Ground State

**Figure 3:**
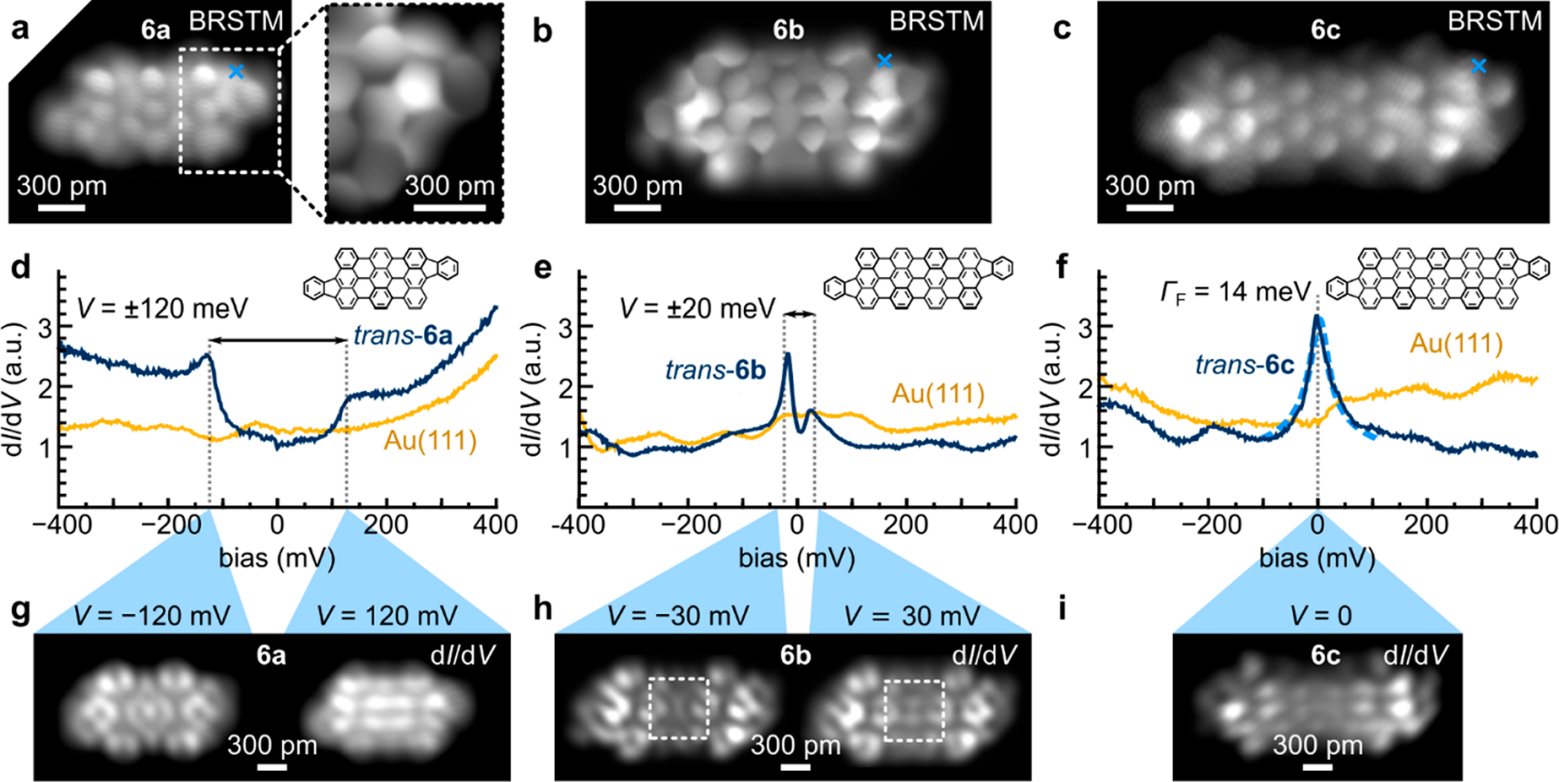
Experimental structure and electronic states of oligoanthenes. (a) BRSTM scans (*V* = 0, *V*_ac_ = 30 mV) of *trans*-**6a** (left), including a close-up BRSTM scan (*V* = 350 mV, *V*_ac_ = 50 mV) of the fluorenyl end (right). (b) BRSTM scan (*V* = 350 mV, *V*_ac_ = 50 mV) of *trans*-**6b**. (c) BRSTM scan (*V* = −300 mV, *V*_ac_ = 100 mV) of *trans*-**6c**. (d) STS d*I*/d*V* spectrum acquired on *trans*-**6a** (*V*_ac_ = 1 mV, location marked in (a)). (e) STS d*I*/d*V* spectrum acquired on *trans*-**6b** (*V*_ac_ = 1 mV, location marked in (b)). (f) STS d*I*/d*V* spectrum acquired on *trans*-**6c** (*V*_ac_ = 1 mV, location marked in (c)). The dashed blue line is a Frota fit to the zero-bias resonance. The Frota width is Γ_F_ = 14 meV. (g) d*I*/d*V* maps (*V* = −120 mV (left) and *V* = 120 mV (right), *V*_ac_ = 20 mV) of *trans*-**6a**. (h) d*I*/d*V* maps (*V* = −30 mV (left) and *V* = 30 mV (right), *V*_ac_ = 4 mV) of *trans*-**6b**. (i) d*I*/d*V* map (*V* = 0, *V*_ac_ = 20 mV) of *trans*-**6c**. d*I*/d*V* maps were taken in constant height mode. All STM measurements were performed at *T* = 4.5 K.

Using five-membered rings to retain the electron occupation of 7-AGNR end states in oligoanthenes **6a**–**d** on Au(111), we can study the intramolecular coupling between end states as a function of molecular length. This was accomplished by characterizing the four oligoanthenes **6a**–**d** by BRSTM, STS, and differential conductance mapping. Representative spectroscopic data for the *trans* isomers of **6a**–**c** are summarized in Figure 3. BRSTM images (Figure 3a–c) confirm the molecular structures of **6a**–**c** including the fluorenyl end groups (Figure 3a inset). d*I*/d*V* point spectroscopy of **6a** shows two distinct step-like spectral features located symmetrically around zero bias at *V* = ±120 mV (Figure 3d). d*I*/d*V* maps at these energies reveal strikingly different orbital patterns (Figure 3g). The *cis* isomer of **6a** shows identical behavior (Figure S4).

Similar electronic behavior is also observed for **6b**, albeit with a markedly reduced spectral gap of Δ*E* = 40 meV (Figure 3e). d*I*/d*V* maps acquired at the peak energies (Figure 3h) reveal a horizontal nodal plane for the higher energy state that is absent in the lower energy state (dashed boxes). Similarly to **6a**, the *cis* isomer of **6b** behaves identically to its *trans* counterpart, with resonant features at the same energies and with similar orbital patterns at these energies (Figure S4).

**6c** is different in that it has no spectral gap but rather exhibits a single peak centered at zero bias (Figure 3f), similarly to **6d**. This implies that **6c** exists in an open-shell ground state where single electrons occupy each of the two end states, and the moments of these electrons are individually Kondo-screened by itinerant electrons in the gold surface. The interaction strength between the electrons in the two SOMOs is thus small compared to the Kondo binding energy *k*_B_*T*_K_. A Frota fit to the zero-bias resonance is shown by the blue dashed line in Figure 3f, and a peak width of Γ_F_ = 14 meV is obtained (corresponding to *T*_K_ ≈ 400 K).^67^ The differential conductance map recorded at zero bias for *trans*-**6c** is shown in Figure 3i. In addition to a zero-bias peak, **6c** also exhibits a positive ion resonance (PIR) at *V* = −550 mV (Figure S5a). Differential conductance maps acquired at this bias reveal an orbital pattern similar to that of maps recorded at zero-bias (Figure S5b), implying that the same SOMOs are probed both at *V* = 0 mV and at *V* = −550 mV. The presence of distinct tunneling pathways into the same SOMOs (i.e., the Kondo peak at zero bias and the PIR at *V* = −550 mV) corroborates the open-shell nature of **6c**. Similar electronic behavior is observed in the *cis* isomer (Figure S4).

### Theoretical Length Dependence of the Ground State

**Figure 4:**
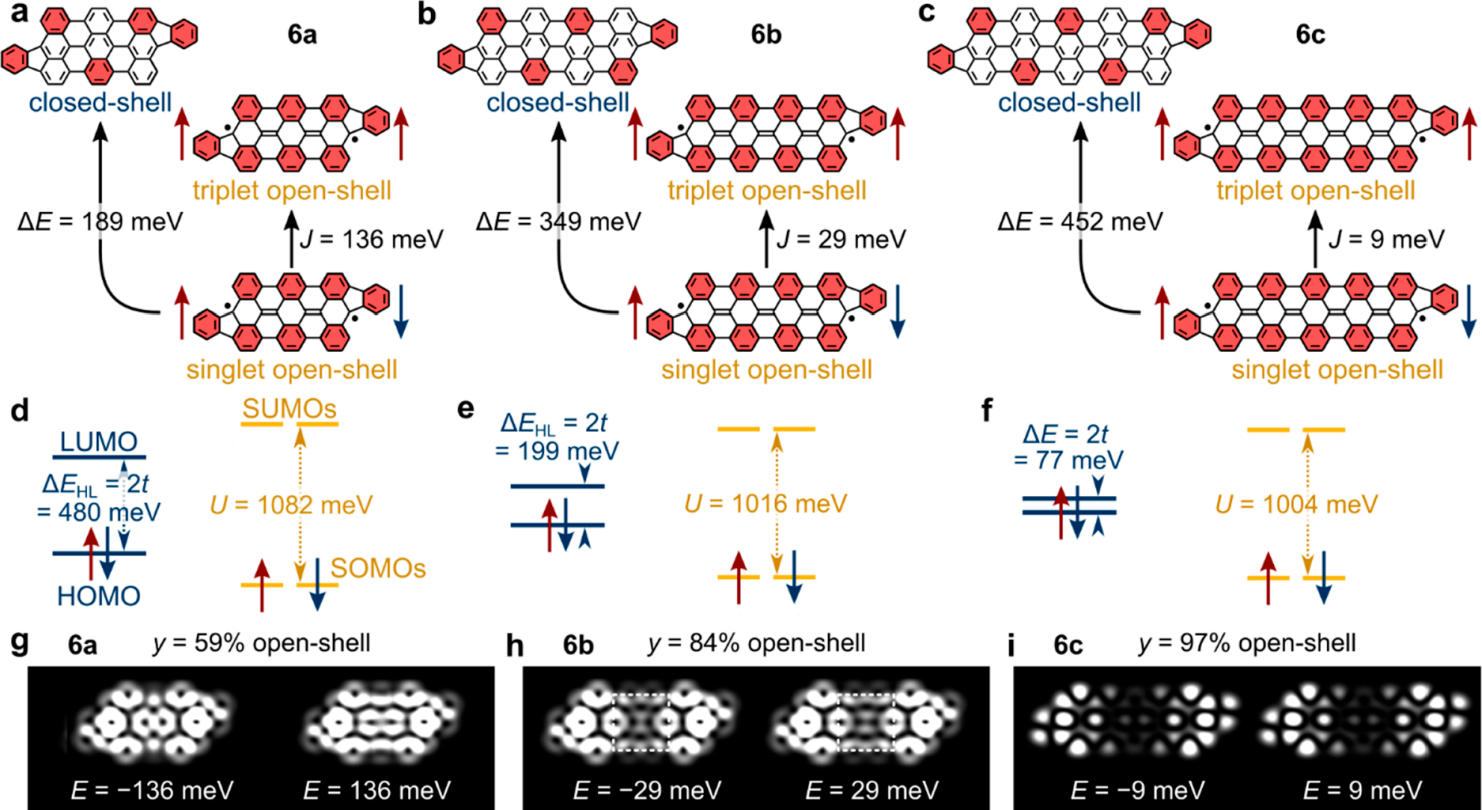
Theoretical analysis of oligoanthenes. (a–c) Kekulé structures corresponding to closed-shell (left) and open-shell (right) configurations for (a) *trans*-**6a**, (b) *trans*-**6b**, and (c) *trans*-**6c**. Clar sextets are highlighted in red. Energy differences between the singlet and triplet open-shell configurations (*J* = *E*_T_ – *E*_S_) and between the closed-shell and singlet open-shell configurations (Δ*E* = *E*_C_ – *E*_S_) as obtained from DFT calculations. (d–f) DFT-calculated HOMO–LUMO gaps (Δ*E*_HL_ = *E*_L_ – *E*_H_) for the closed-shell configurations of *trans*-**6a**–**c** and Coulomb gaps between SOMOs and SUMOs (*U* = *E*_SUMO_ – *E*_SOMO_) for the open-shell configurations of *trans*-**6a**–**c** indicated in blue and yellow, respectively. The energy levels are referenced to their midgap energy (set at *E* = 0). (g–i) Simulated d*I*/d*V* maps of the frontier states of (g) *trans*-**6a**, (h) *trans*-**6b**, and (i) *trans*-**6c**, using weighted superpositions of the HOMO and LUMO from the closed-shell configuration and SOMOs from the open-shell configuration using biradical indices *y* as indicated.

Our experiments imply that the longer oligoanthenes (**6c** and **6d**) exhibit open-shell character with the spins on either end of the molecules individually Kondo-screened. This is reasonably intuitive, since the coupling between end states (characterized by the hybridization interaction parameter *t*) should decrease with increasing separation between them. Analogously, the aromatic stabilization gained by the increased number of Clar sextets in the open-shell or biradical resonance structure exceeds the energy required to break one π-bond and generate two radicals (Figure 4a–c).^32^ For the shorter oligoanthenes (**6a** and **6b**), the situation is more complicated. The symmetric spectral features around zero bias and step-like nature of these features in **6a** are suggestive of spin-flip excitations for two exchange-coupled spins from a singlet ground state to a triplet excited state, similarly to the previously studied Clar goblet system.^46^ However, the different wave function patterns observed at the edges of the pseudogap (Figure 3g,h) suggest the quite different scenario of tunneling into the highest occupied molecular orbital (HOMO) and lowest unoccupied molecular orbital (LUMO) of a closed-shell configuration resulting from strong hybridization between the two end states.

These two apparently contradictory observations can be reconciled by considering that shorter oligoanthenes exhibit a multireference ground state that has contributions from both open-shell and closed-shell configurations.^51−53,68,69^ The partial open-shell nature of the ground state causes nonzero local magnetic moments to exist on opposite sides of the NG, allowing inelastic spin-flip excitations to be recorded in d*I*/d*V* spectroscopy.^29,70,71^ This explains the experimentally obtained spectral line shape with symmetrically distributed features around zero bias. However, depending on the polarity of the tunneling bias, different hybridized combinations of end states—bonding and antibonding—can also be probed. This is because the GNR exhibits partial open- and closed-shell character, and as a result, tunneling electrons have both an elastic channel (involving the HOMO and LUMO) and an inelastic channel (involving the SOMOs) available. STM spectroscopic signatures thus naturally contain a combination of both features.^72^

The mixed open-shell/closed-shell character of this system can be modeled using the Hubbard dimer model (HDM), similar to the procedure of Ortiz et al.^61^ where the results from *ab initio* density functional theory (DFT) calculations are used as input parameters. Our DFT calculations are at the single-determinant level (as opposed to explicit multireference models such as configuration-interaction (CI) or complete active space (CAS) calculations^68^), and so we separately simulated the closed-shell, singlet open-shell, and triplet open-shell configurations of the oligoanthenes in vacuo. Figure 4a–c shows the calculated energy difference Δ*E* = *E*_C_ – *E*_S_ between the closed-shell ground state energy *E*_C_ and the singlet open-shell ground state energy *E*_S_ for *trans* oligoanthenes, as well as the exchange coupling *J* (equal to the energy difference between the singlet and triplet (*E*_T_) open-shell configuration energies; *J* = *E*_T_ – *E*_S_) and the Coulomb gap *U* (equal to the energy difference between the SOMOs and SUMOs in the singlet open-shell configuration). The calculated *J* values (Figure 4a–c) agree reasonably well with the energies of the spectral features observed for **6a** and **6b** (Figure 3d,e), suggesting that spin-flip excitations play a role in the spectroscopic features that we observe for these oligomers. We also calculated the HOMO–LUMO gaps from closed-shell calculations (Figure 4d–f, blue), enabling us to parametrize the hybridization interaction between end states as Δ*E*_HL_ = *E*_LUMO_ – *E*_HOMO_ = 2*t*.

We used our DFT results as input for a HDM calculation where the parameters *t* and *J* allow calculation of the biradical index *y* (the procedure is described in Supplementary Discussion 3). In the two-site Hubbard model, the ground state of each oligoanthene is a weighted sum of open-shell singlet and closed-shell singlet, and the first excited state is a triplet. We thus conclude that the observed spectral features in STS indeed include singlet-to-triplet spin-flip excitations. The HDM calculation suggests that the oligoanthenes exhibit a smooth evolution to increased open-shell character as the length is increased. This overall physical picture suggests that d*I*/d*V* maps should include both a fraction of closed-shell wave function and a fraction of open-shell wave function as determined by the HDM *y* parameter. If we follow this protocol and use the *y*-values obtained from our HDM calculation (75% ≤ *y* ≤ 99% for structures **6a**–**c**) then we obtain a poor fit to our experimental d*I*/d*V* maps because the *y*-values are too high (see Supplementary Figure S6). We do, however, obtain reasonable fits to the data for reduced *y*-values of 59%–97% for structures **6a**–**c**, as shown in Figure 4g–i.^51,69,73^ This suggests that neglect of the substrate in our calculations leads to an overestimation of the open-shell proportion of the low-lying states.

## Conclusions

7-AGNR segments appear to exhibit partial closed-shell and partial open-shell character on Au(111), and their open-shell character increases in a smooth evolution as the length is increased. **6a** has the highest closed-shell character, with **6b** being more open-shell and **6c**,**d** having end states that are so weakly hybridized that they are individually Kondo-screened by electrons in the gold surface.^18^ This progression is characterized by a decrease in the exchange-coupling strength *J* between spins on opposite ends of the 7-AGNR: *J* = 120 meV for **6a**, *J* = 20 meV for **6b**, and *J* < *k*_B_*T*_K_ for **6c** and **6d**. The overall trend, including spin-flip excitations, is captured by combining DFT calculations with the HDM, but the proportion of open-shell character appears to be overestimated. We assume that the omission of the metallic substrate in the DFT calculations causes inaccuracies in the singlet–triplet energies because of the absence of both charge screening and magnetic (i.e., Kondo) screening.^71^ Additionally, DFT may underestimate HOMO–LUMO gaps, and this may also impact the value of the hybridization parameter *t* and, by extension, the value of the biradical index *y*. These inaccuracies may explain the underestimation of the degree of open-shell character in the DFT-HDM model.

Although we are unable to mitigate the effects of substrate-induced screening, we successfully countered the unwanted effect of surface-induced charge transfer (which quenches magnetism). This was made possible by fusing five-membered rings to GNR zigzag ends, which has the benefit of inducing a downward shift in the end state energy due to the electron-accepting character of the cyclopentadienyl ring, thereby counteracting the p-doping effect of the gold surface. Tuning local mode energy offsets is thus a useful tool for quantum engineering of NGs.

## Methods/Experiments

### Synthetic Procedures

Unless otherwise stated, all manipulations of air- and/or moisture-sensitive compounds were carried out in oven-dried glassware under an atmosphere of N_2_. All solvents and reagents were purchased from Alfa Aesar, Spectrum Chemicals, Acros Organics, TCI America, and Sigma-Aldrich and used as received unless otherwise noted. Organic solvents were dried by passing through a column of alumina and were degassed by vigorous bubbling of N_2_ through the solvent for 20 min. Flash column chromatography was performed on SiliCycle silica gel (particle size 40–63 μm). Thin-layer chromatography was carried out using SiliCycle silica gel 60 Å F-254 precoated plates (0.25 mm thick) and visualized by UV absorption. All ^1^H and ^13^C NMR spectra were recorded on a Bruker AV-600 spectrometer and are referenced to residual solvent peaks (CDCl_3_, ^1^H NMR = 7.26 ppm, ^13^C NMR = 77.16 ppm; CD_2_Cl_2_, ^1^H NMR = 5.32 ppm, ^13^C NMR = 53.84 ppm). EI mass spectrometry was performed on an AutoSpec Premier (Waters) system in positive ionization mode. MALDI mass spectrometry was performed on a Voyager-DE PRO (Applied Biosystems Voyager System 6322) instrument in positive mode using a matrix of dithranol. 10,10′-Dibromo-9,9′-bianthryl was synthesized following a reported procedure.^74^

### Preparation of MAD Transfer Samples

Samples of **1a**–**d** were mixed with solid pyrene at *T* = 24 °C in a scintillation vial to make a 0.1 wt % mixture of sample in pyrene. The solid mixtures were placed into a preheated sand bath held at *T* = 200 °C until the pyrene completely melted (approximately 2 min). The melted mixtures were lightly swirled for 15 s in the sand bath to ensure homogeneous dispersion of the sample in the pyrene melt. The melted mixtures were immediately placed in an *T* = −78 °C acetone/dry ice bath to induce rapid crystallization. The solid was ground to a fine powder prior to deposition.

### Sample Preparation

Clean Au(111) surfaces were prepared through iterative cycles of Ar^+^ sputtering (*p* = 4 × 10^–6^ Torr) and annealing (*T* = 400 °C). The fiberglass applicator of the MAD transfer setup was outgassed in high vacuum (*p* ≈ 5 × 10^–8^ Torr) by resistive heating of a tungsten filament to approximately *T* = 500 °C for 20 min prior to use.^63^ After cooldown, the chamber was vented, and the applicator was removed and lightly pressed into the finely ground MAD transfer sample. The loaded fiberglass applicator was reintroduced to the chamber and pumped down to a high vacuum. After a high vacuum was reached, a cleaned Au(111) substrate was transferred into the chamber, and the fiberglass applicator was pressed against the Au(111) substrate to transfer the sample material.

### STM Measurements

All STM experiments were performed using a commercial Createc LT-STM instrument operating at *T* = 4.5 K using chemically etched tungsten STM tips. d*I*/d*V* spectra were recorded using a lock-in amplifier with a modulation frequency of *f* = 533 Hz and a modulation amplitude of *V*_ac_ = 2.0 mV. All STS experiments and differential conductance maps were acquired in constant height mode. Image processing of the STM scans was performed using WSxM software.^75^ Tip passivation with carbon monoxide or other surface absorbates was achieved using standard methods.^76^

### Calculations

First-principles calculations were performed at the density functional theory level as implemented in VASP.^77^ We adopted the hybrid HSE06^78^ functional for both structure relaxations and accurate band gap evaluation. Electron–core interactions were described through the projector augmented wave (PAW) method.^79,80^ Kohn–Sham wave functions were expanded in a plane wave basis set with a cutoff on kinetic energy of 400 eV. All structures were subject to periodic boundary conditions with a vacuum layer of 10 Å in all directions to prevent interaction between replica images. Atomic positions were optimized using the conjugate gradient method, where the total energy and atomic forces were minimized. The convergence criterion for energy was chosen to be 10^–6^ eV, and the maximum force acting on each atom was less than 0.02 eV/Å. Open-shell configurations were obtained by setting appropriate initial magnetic moments and conducting spin-resolved calculations.

Additional DFT calculations involving semi-infinite leads as displayed in Supplementary Figure S1 were performed within the generalized gradient approximation to the exchange and correlation functional following Perdew, Burke, and Ernzerhof^81^ as implemented in SIESTA.^82^ Core electrons were described by separable norm-conserving pseudopotentials,^83^ whereas single-particle wave functions of valence electrons were expanded as linear combinations of atomic orbitals with double-ζ polarization quality. Real space integrations were performed with a 400 Ry mesh cutoff, and a *k*-point grid of 150 points in the semi-infinite directions was utilized.

## Supporting Information Available

Discussion of the low-energy emergent physics in NGs, the molecular orbital perspective of off-zero modes and the Hubbard dimer model, synthetic procedures and schemes, ^1^H and ^13^C{^1^H} NMR spectra, experimental results on the *cis* and *trans* isomers of oligoanthenes, experimental results on the identification of the PIR in **6c**, and simulated d*I*/d*V* maps (PDF)
