# Supplementary material for: Five-Membered Rings Create Off-Zero Modes in Nanographene
Source: ACS Nano. 2023 Dec 5;17(24):24901–9. doi: 10.1021/acsnano.3c06006 (PMC10753889; doi:10.1021/acsnano.3c06006)
Supplement: Supplementary file 1 — nn3c06006_si_001.pdf [file nn3c06006_si_001.pdf]

Supporting Information for

Five-membered rings create off-zero modes in nanographene

Peter H. Jacobse[†‖#], *Michael C. Daugherty*[‡#], *Kristiāns Čerņevičs*[§^#], *Ziyi Wang*[†‖∇‡], *Ryan D. McCurdy*[‡], *Oleg V. Yazyev*[§^*], *Felix R. Fischer*[‡‖∇◊*], *Michael F. Crommie*[†‖∇*]

†Department of Physics, University of California, Berkeley, CA 94720, U.S.A.

‡Department of Chemistry, University of California, Berkeley, CA 94720, U.S.A.

§Institute of Physics, Ecole Polytechnique Fédérale de Lausanne (EPFL), 1015, Lausanne, Switzerland

‖Materials Sciences Division, Lawrence Berkeley National Laboratory, Berkeley, CA 94720, U.S.A.

∇Kavli Energy NanoSciences Institute at the University of California Berkeley and the Lawrence Berkeley National Laboratory, Berkeley, California 94720, U.S.A.

◊Bakar Institute of Digital Materials for the Planet, Division of Computing, Data Science, and Society, University of California, Berkeley, CA 94720, USA.

S3 Supplementary Discussion 1: Understanding low-energy emergent physics in NGs
S4 Supplementary Discussion 2: Molecular orbital perspective of off-zero modes
S5 **Figure S1.** Off-zero modes versus zero-modes.
S6 Supplementary Discussion 3: Hubbard dimer model
S9 Synthetic Procedures
Scheme S1. Synthesis of **1a** and **1b**.
S11 **Scheme S2.** Synthesis of **1c** and **1d**.
S13 **Figure S2.** ^{1}H NMR (600 MHz, CD_2Cl_2) of **7** at 24 °C.
S14 **Figure S3.** $^{13}C\{^{1}H\}$ NMR (151 MHz, $CDCl_3$) of **7** at 24 °C.
S15 **Figure S4.** Experimental study of the structure and ground state of oligoanthenes.
Figure S5. Identification of the positive ion resonance in **6c**.
S16 **Figure S6.** Simulated dI/dV maps for frontier states with s-wave and p-wave tips.
S17 Supplementary references

Supplementary Discussion 1: Understanding low-energy emergent physics in NGs

Successful application of strategies to create designer quantum structures in NGs involves understanding the electronic structure at two distinct levels of abstraction. First, the emergence of zero-modes with wave functions $|\psi_i>$ happens at the atomistic level from considerations regarding topology, heteroatoms, and the sublattice structure.[1–3] Once the zero-modes are established at this level, their subsequent coupling can be understood at an effective level by taking all the emergent zero-mode wave functions as the basis for a more "coarse-grained" Hubbard model, where they are coupled through interaction integrals t_{ij} (the hopping between zero-mode *i* and *j*) and are subject to on-site electron-electron repulsions described by an effective Hubbard parameter *U*.[4] These two levels of abstraction simplify the challenge of engineering desired electronic and magnetic properties into NGs by dividing it into two straightforward challenges: 1) creation of an appropriate basis of low-energy basis states and 2) defining and solving the Hamiltonian arising from interactions within the effective basis.[4]

The tuning of the effective coupling *t* between zero-modes has caught significant attention in recent years: it has been shown that the sign and magnitude of *t* depends – often non-trivially – on the physical separation of zero-modes, the bandgap and width of the hosting NG, and the intricate overlap pattern of the zero-mode wave functions. When the effective coupling *t* is strong, zero-modes hybridize into bonding and antibonding combinations. When the effective coupling *t* is negligible, zero-modes remain separated as non-interacting localized states. When *t* is weak compared to the Hubbard *U*, but not negligible, zero-modes that are singly occupied can cause electron spins to assume a correlated spin ground state, for example a singlet (antiferromagnetic) or triplet (ferromagnetic) ground state in the case of two interacting spins. A parameter in the Hubbard model that has caught much less attention than *t* is the on-site (or binding) energy ε_i. Whereas playing with staggered hopping parameters (t_1 and t_2) has permitted the creation of Su-Schrieffer-Heeger-type chains,[5] quantum structures with staggered on-site energies (that is, ε_1 and ε_2) have not yet been explored. The reason is that – in contrast with *t* – methods to tailor ε are still missing from the quantum engineering toolkit. For this reason, off-zero modes are appealing targets.

Supplementary Discussion 2: Molecular orbital perspective of off-zero modes

The off-zero modes generated by five-membered rings can be understood from a molecular orbital perspective by comparing them with zero-modes. Zero-mode wave functions in NGs (or in general, on any bipartite lattice) can intuitively be understood as wave functions with a wavelength of 4 lattice constants (4a), or equivalently a "Fermi" momentum of $k_F = 2\pi/(4a)$. Typically, they are sublattice-polarized with alternating phase on one sublattice and vanishing density on the other sublattice. Two systems that can fit a zero-mode wave function are cyclobutadiene (which features a "4 site loop"), and the zigzag end of a 7-AGNR (which features a "16 site loop"), as shown in Supplementary Figure S1a. Here, the plus and minus signs indicate the phase of the wave function, and "0" indicates that the wave function has vanishing density. In both cyclobutadiene and the 7-AGNR end, the zero-mode wave functions can be interpreted as a superposition of "particle on a ring"-type plane waves counterpropagating in the clockwise and counterclockwise directions with electron momentum $k = 2\pi/(4a)$. Such a 4-site periodic plane wave does not fit on a five-membered ring, and so a zero-mode cannot exist on it. In order to make the wave function satisfy periodic boundary conditions on the cyclopentadienyl ring, the momentum needs to be decreased to $k' = 2\pi/(5 \text{ sites})$ (so that the (unnormalized) wave function expansion coefficients become the quintic roots of unity), as shown in Supplementary Figure S1b. As the electron momentum is decreased, the energy is correspondingly lowered – from the zero-mode energy to the off-zero mode energy.

The intuition gained by the above analysis suggests that the link between five-membered rings and off-zero modes is general. Indeed, five-membered rings may be used paradigmatically to engineer off-zero modes much in the same way as Ovchinnikov's rule can be used to engineer zero-modes. In the case of fluorenyl caps, the zero-mode originally extant on the zigzag edge is lowered in energy as it bleeds into the five-membered ring. When a five-membered ring is attached to an armchair edge, on the other hand, the new state that is generated corresponding to the unpaired atom becomes a new zero-mode. This is conceptualized in Supplementary Figure S1c, d.

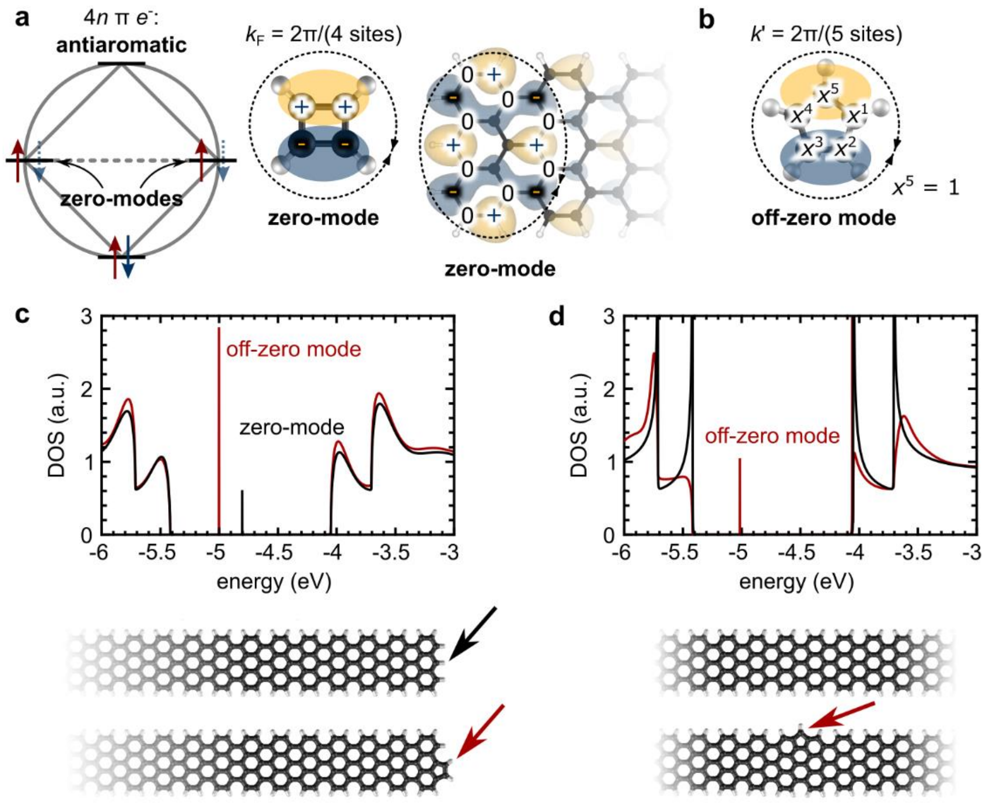

Figure S1. Off-zero modes versus zero-modes. (a) A frost circle of cyclobutadiene (left), a cyclobutadiene frontier orbital (center) with phase of the wave function (+ or –) indicated and a 7-AGNR end state (right) with phase of the wave function indicated. A λ = 4-site loop can be recognized in the cyclobutadiene orbital, and a 4λ = 16-site loop (so λ = 4 sites) can be recognized in the 7-AGNR end state. (b) A frontier orbital of the cyclopentadienyl radical, with unnormalized expansion coefficients of the wave function on the atomic sites indicated. Here, the off-zero mode orbital corresponds to a λ = 5-site loop, which has lower momentum k and lower energy E than a zero-mode orbital. (c) DFT-calculated DOS of a semi-infinite 7-AGNR (black) and its pentagon-capped analog (red). The structures are shown below the DOS. (d) DFT-calculated DOS of an infinite 7-AGNR (black) and its analog with one pentagon attached to the side (red). The structures are shown below the DOS.

Supplementary Discussion 3: Hubbard dimer model

Taking the SOMOs localized on the left- and right ends of the oligoanthenes as the single-particle basis states, a complete active space describing all possible two-electron configurations can be written as[6,7]

$$|\psi\rangle = c_1|\uparrow\downarrow,0\rangle + c_2|0,\uparrow\downarrow\rangle + h_1|\uparrow,\uparrow\rangle + h_2|\downarrow,\downarrow\rangle + l_1|\uparrow,\downarrow\rangle + l_2|\downarrow,\uparrow\rangle, \quad \text{(S1)}$$

where c_1, c_2 are expansion coefficients for closed-shell states, h_1 and h_2 for high-spin states (S_z = 1), l_1 and l_2 for low-spin states (S_z = 0), and the first entry in each ket denotes the left-localized basis state (or state a) while the second entry denotes the right-localized basis state (or state b). Application of the Hubbard Hamiltonian

$$\hat{H} = t\sum_{\sigma}\left(a_{\sigma}^{\dagger}b_{\sigma} + b_{\sigma}^{\dagger}a_{\sigma}\right) + U(n_{a\uparrow}n_{a\downarrow} + n_{b\uparrow}n_{b\downarrow}) + g\mu_B\sum_{\sigma,\sigma'}\boldsymbol{B}\cdot\boldsymbol{S}_{\sigma,\sigma'}\left(a_{\sigma}^{\dagger}a_{\sigma'} + b_{\sigma}^{\dagger}b_{\sigma'}\right) \quad \text{(S2)}$$

to this wavefunction gives six coupled equations that can be written in matrix form giving the Hamiltonian matrix

$$\mathbf{H} = \begin{pmatrix} U & 0 & 0 & 0 & -t & t \\ 0 & U & 0 & 0 & -t & t \\ 0 & 0 & g\mu_B B_z & 0 & 0 & 0 \\ 0 & 0 & 0 & -g\mu_B B_z & 0 & 0 \\ -t & -t & 0 & 0 & 0 & 0 \\ t & t & 0 & 0 & 0 & 0 \end{pmatrix} \quad \text{(S3)}$$

Diagonalization of this Hamiltonian gives the following solutions:

- Three triplet states centered around zero that may be split by the Zeeman effect in an external magnetic field $\boldsymbol{B}$:

$$E_{\mathrm{T},1} = g\mu_B B_z; \qquad |\psi\rangle_{\mathrm{T},1} = |\uparrow,\uparrow\rangle$$

$$E_{\mathrm{T},0} = 0; \qquad |\psi\rangle_{\mathrm{T},0} = \frac{|\uparrow,\downarrow\rangle + |\uparrow,\downarrow\rangle}{\sqrt{2}}$$

$$E_{\mathrm{T},-1} = -g\mu_B B_z; \qquad |\psi\rangle_{\mathrm{T},-1} = |\downarrow,\downarrow\rangle \quad \text{(S4)}$$

- One pure closed-shell singlet separated from the spin-triplet by the Coulomb energy U:

$$E_{\mathrm{C}-} = U; \qquad |\psi\rangle_{\mathrm{C}-} = \frac{|0,\uparrow\downarrow\rangle - |\uparrow\downarrow,0\rangle}{\sqrt{2}} \quad \text{(S5)}$$

- Two singlet states with partial open-shell and partial closed-shell character:

$$E_{\mathrm{S}-} = \frac{U}{2} - \sqrt{\left(\frac{U}{2}\right)^2 + (2t)^2}; \qquad |\psi\rangle_{\mathrm{S}-} = u|\psi\rangle_{\mathrm{C}+} + v|\psi\rangle_{\mathrm{O}}$$

$$E_{\mathrm{S+}} = \frac{U}{2} + \sqrt{\left(\frac{U}{2}\right)^2 + (2t)^2}; \qquad |\psi\rangle_{\mathrm{S-}} = w|\psi\rangle_{\mathrm{C+}} + x|\psi\rangle_{\mathrm{O}}, \tag{S6}$$

where *u* and *v*, *w* and *x* are expansion coefficients that have a functional dependence on *t* and *U*, and where

$$|\psi\rangle_{\mathrm{C+}} = \frac{|\uparrow\downarrow,0\rangle + |0,\uparrow\downarrow\rangle}{\sqrt{2}}; \qquad |\psi\rangle_{\mathrm{O}} = \frac{|\downarrow,\uparrow\rangle - |\uparrow,\downarrow\rangle}{\sqrt{2}} \tag{S7}$$

are two-particle wavefunctions with pure closed-shell character and with pure open-shell character, respectively.

It can readily be seen that the lowest-energy state, or ground state, of the Hubbard dimer model is the spin-singlet at energy $E_{\mathrm{S-}}$. In the absence of Zeeman splitting, this level is separated from the spin-triplet by a singlet-triplet energy difference

$$J = \sqrt{\left(\frac{U}{2}\right)^2 + (2t)^2} - \frac{U}{2} = \sqrt{\left(\frac{U}{2}\right)^2 + \Delta E_{\mathrm{HL}}^2} - \frac{U}{2} \approx \frac{4t^2}{U} - \frac{16t^4}{U^3} + \cdots, \tag{S8}$$

where the right-hand side shows the Taylor expansion of the square root, which may be accurate for $U > t$, but which converges poorly for small U/t and is therefore not applicable in the regime of interest.

With values of *U* and *t* for each oligoanthene in hand, the weights *u* and *v* of the singlet ground state were now calculated by numerical diagonalization of the Hamiltonian **H**. The projection P_2 of the wave function on the closed shell basis functions was calculated as

$$P_2 = |\langle\psi|_{\mathrm{C+}}\psi\rangle_{\mathrm{S-}}|^2, \tag{S9}$$

and it was verified that P_2 runs from 0.5 to 0 as U/t increases from 0 to infinity. The percentage biradical character is obtained through $y = 1 - 2P_2$. Using this procedure, we calculated the following HDM-derived values of the biradical index *y*: *y* = 0.75 (**6a**), *y* = 0.93 (**6b**), *y* = 0.99 (**6c**) and *y* = 1.00 (**6d**).

Finally, the biradical index *y* was used to construct weighted sums of simulated closed-shell d*I*/d*V* maps and open-shell d*I*/d*V* maps. Here we first calculated the experimental d*I*/d*V* maps corresponding to the HOMO and LUMO from the single-particle wavefunctions obtained from the closed-shell DFT calculations. We then constructed the single-particle wavefunctions of the SOMOs by linear superpositions of the HOMO and LUMO wavefunctions:

$$|\psi\rangle_{\mathrm{SOMO}s} = \frac{|\psi\rangle_{\mathrm{HOMO}} \pm |\psi\rangle_{\mathrm{LUMO}}}{\sqrt{2}} \tag{S9}$$

The resulting maps, calculated for all oligoanthenes and for both s-wave and p-wave (carbon monoxide passivated) tips, are shown in Supplementary Figure S6. We added the maps corresponding to the SOMOs and HOMO and LUMO together with weights of y and $(1 - y)$, respectively, to construct the simulated maps in Figure 4g–i.

Synthetic Procedures

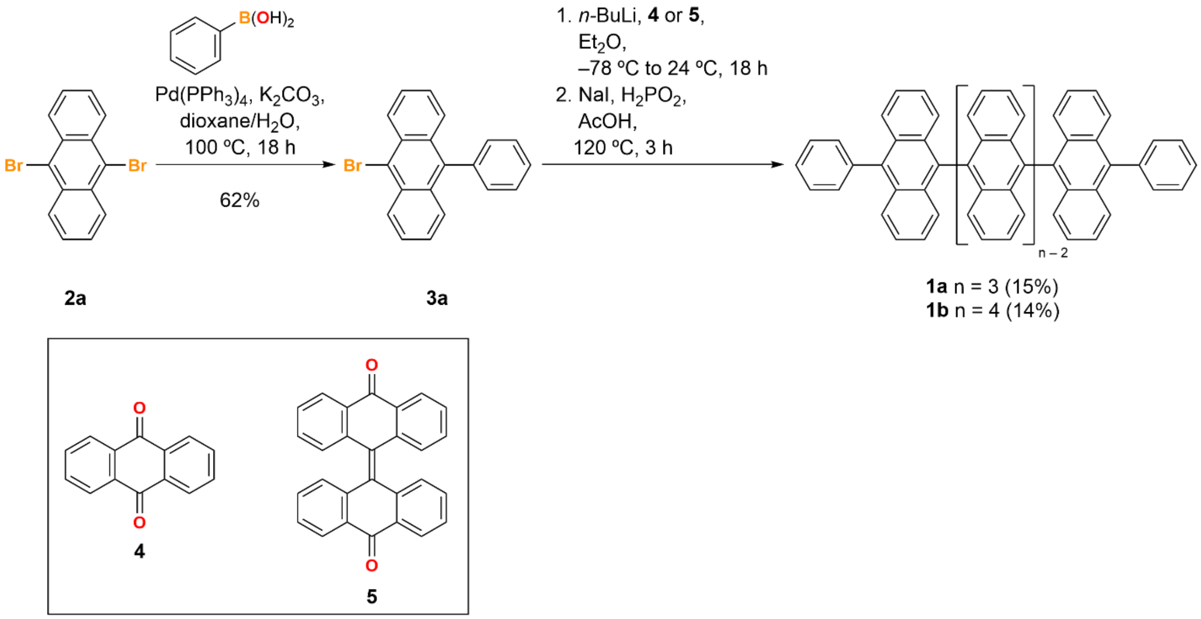

Scheme S1. Synthesis of **1a** and **1b**.

9-bromo-10-phenylanthracene (**3a**): A 500 mL Schlenk flask was charged with 9,10-dibromoanthracene (**2a**, 1.00 g, 3.00 mmol), phenylboronic acid (0.37 g, 3.00 mmol), and potassium carbonate (2.50 g, 18.0 mmol) in dioxane (180 mL) and H_2O (30 mL). The reaction mixture was degassed by sparging with N_2 for 20 min before tetrakis(triphenylphosphine)palladium(0) (0.35 g, 0.300 mmol) was added under N_2. A reflux condenser was attached and the reaction mixture was stirred under N_2 for 18 h at 100 °C. The reaction mixture was concentrated on a rotary evaporator, diluted with H_2O (200 mL), and extracted with CH_2Cl_2 (400 mL). The combined organic phases were washed with H_2O (100 mL) and saturated aqueous NaCl (100 mL), dried over $MgSO_4$, and concentrated on a rotary evaporator. Column chromatography (SiO_2; hexanes) yielded **3a** (0.619 g, 1.86 mmol, 62 %) as a light yellow solid. ^{1}H NMR (600 MHz, CD_2Cl_2) δ = 8.60 (d, J = 8.8 Hz, 2H), 7.68–7.54 (m, 7H), 7.43–7.37 (m, 4H) ppm. (Analysis matches reported values)[8]

10,10''-diphenyl-9,9':10',9''-teranthracene (**1a**): An oven dried 100 mL Schlenk flask was charged with **3a** (0.100 g, 0.300 mmol) in anhydrous Et_2O (4 mL) under N_2. The yellow suspension was cooled to –78 °C and n-BuLi (0.18 mL, 0.45 mmol, 2.5 M in hexanes) was added dropwise. The

suspension was stirred for 2 h at –78 °C and added via cannula to a suspension of anthraquinone (**4**, 0.021 g, 0.10 mmol) in anhydrous Et_2O (3 mL) at 0 °C. The reaction mixture was stirred at 0 °C for 1 h and then warmed to 24 °C for an additional 18 h. The suspension was quenched with AcOH (3 mL) and the precipitate was filtered and washed with Et_2O. The solid precipitate was added to a 200 mL Schlenk flask with NaI (0.180 g, 1.20 mmol) and $NaH_2PO_2 \cdot H_2O$ (0.191 g, 1.80 mmol). The flask was evacuated and backfilled with N_2 three times, degassed AcOH (5 mL) was added, and the reaction mixture was stirred at 120 °C for 3 h under N_2 and the exclusion of light. The orange suspension was cooled to 24 °C, and the solid was filtered and washed with H_2O and MeOH to afford **1a** (0.010 g, 0.015 mmol, 15%) as a yellow solid. HRMS (MALDI-TOF) *m/z*: $[C_{54}H_{34}]^+$, calcd. $[C_{54}H_{34}]$ 682.2661; found 682.6091. The solubility of **1a** in common deuterated solvents is insufficient to acquire ^1H or $^{13}C\{^1H\}$ NMR spectra.

10,10'''-diphenyl-9,9':10',9'':10'',9'''-quateranthracene (**1b**): An oven dried 10 mL Schlenk flask was charged with **3a** (0.067 g, 0.20 mmol) in anhydrous Et_2O (1.5 mL) under N_2. The yellow suspension was cooled to –78 °C and *n*-BuLi (0.10 mL, 0.25 mmol, 2.5 M in hexanes) was added dropwise. The suspension was stirred for 2 h at –78 °C and added via cannula to a suspension of bianthrone (0.035 g, 0.091 mmol) in anhydrous Et_2O (1.5 mL) at 0 °C. The reaction mixture was stirred at 0 °C for 1 h then warmed to 24 °C for an additional 18 h. The suspension was quenched with AcOH (0.5 mL) and the precipitate was filtered and washed with Et_2O. The solid precipitate was added to a 10 mL Schlenk tube with NaI (0.052 g, 0.34 mmol) and $NaH_2PO_2 \cdot H_2O$ (0.059 g, 0.56 mmol). The tube was evacuated and backfilled with N_2 three times, degassed AcOH (3 mL) was added, and the reaction mixture was sealed under N_2 and stirred at 120 °C for 3 h under the exclusion of light. The orange suspension was cooled to 24 °C, and the solid was filtered and washed with H_2O, Et_2O, AcOH, hexanes, CH_2Cl_2, and pentane to give **1b** (2.5 mg, 0.0029 mmol, 14%) as a yellow solid. HRMS (MALDI-TOF) *m/z*: $[C_{68}H_{42}]^+$, calcd. $[C_{68}H_{42}]$ 858.3287; found 858.2011. The solubility of **1b** in common deuterated solvents is insufficient to acquire ^1H or $^{13}C\{^1H\}$ NMR spectra.

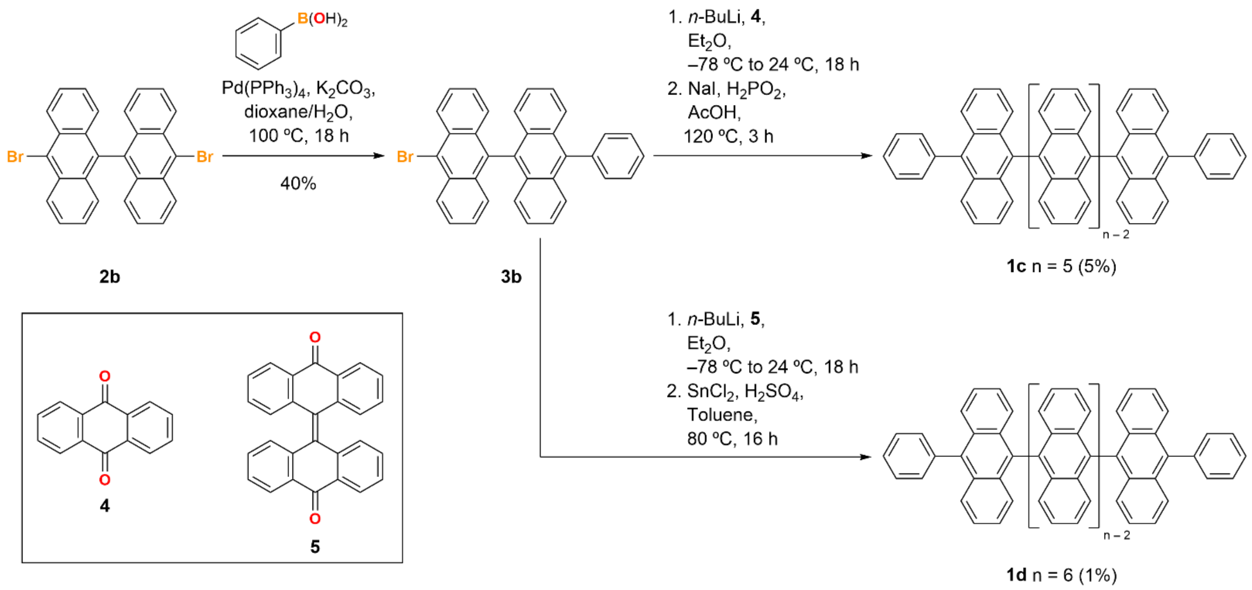

Scheme S2. Synthesis of **1c** and **1d**.

10-bromo-10'-phenyl-9,9'-bianthracene (**3b**): A 500 mL Schlenk flask was charged with **2b** (1.54 g, 3.00 mmol), phenylboronic acid (0.37 g, 3.00 mmol), and potassium carbonate (2.50 g, 18.1 mmol) in dioxane (180 mL) and H_2O (30 mL). The reaction mixture was degassed by sparging with N_2 for 20 min before tetrakis(triphenylphosphine)palladium(0) (0.35 g, 0.300 mmol) was added under N_2. A reflux condenser was attached and the reaction mixture stirred under N_2 for 18 h at 100 °C. The reaction mixture was concentrated on a rotary evaporator, diluted with H_2O (200 mL), and extracted with CH_2Cl_2 (400 mL). The combined organic phases were washed with H_2O (100 mL), saturated aqueous NaCl (100 mL), dried over $MgSO_4$, and concentrated on a rotary evaporator. Column chromatography (SiO_2; hexanes) yielded **3b** (0.600 g, 1.20 mmol, 40 %) as a light yellow solid. ^{1}H NMR (600 MHz, CD_2Cl_2) δ = 8.73 (d, J = 8.0 Hz, 2H), 7.81 (d, J = 8.0 Hz, 2H), 7.69 (m, 2H), 7.62 (m, 5H), 7.33 (m, 2H), 7.25 (m, 4H), 7.15 (m, 2H), 7.08 (m, 2H) ppm; ^{13}C{^{1}H} NMR (151 MHz, $CDCl_3$) δ = 139.1, 138.4, 134.4, 132.6, 132.6, 131.6, 131.3, 130.7, 130.2, 128.6, 128.2, 127.8, 127.6, 127.4, 127.4, 126.9, 126.3, 125.9, 125.4, 123.7; HRMS (EI-TOF) m/z: $[C_{34}H_{21}Br]^+$, calcd. $[C_{34}H_{21}Br]$ 508.0827; found 508.0819.

10,10''''-diphenyl-9,9':10',9'':10'',9''':10''',9''''-quinqueanthracene (**1c**): An oven dried 100 mL Schlenk flask was charged with **7** (0.250 g, 0.491 mmol) in anhydrous Et_2O (6 mL) under N_2. The yellow suspension was cooled to –78 °C and n-BuLi (0.30 mL, 0.74 mmol, 2.5 M in hexanes) was added dropwise. The suspension was stirred for 2 h at –78 °C and added via cannula to a suspension

of anthraquinone (0.050 g, 0.24 mmol) in anhydrous Et_2O (6 mL) at 0 °C. The reaction mixture was stirred at 0 °C for 1 h then warmed to 24 °C for an additional 18 h. The suspension was quenched with AcOH (3 mL) and the precipitate was filtered and washed with Et_2O. The solid precipitate was added to a 200 mL Schlenk flask with NaI (0.202 g, 1.35 mmol) and $NaH_2PO_2 \cdot H_2O$ (0.212 g, 2.00 mmol). The flask was evacuated and backfilled with N_2 three times, degassed AcOH (8 mL) was added and the reaction mixture was stirred at 120 °C for 3 h under N_2 and the exclusion of light. The orange suspension was cooled to 24 °C and the solid was filtered, washed with H_2O, and MeOH to give **1c** (0.012 g, 0.011 mmol, 5%) as a yellow solid. HRMS (MALDI-TOF) *m/z*: $[C_{82}H_{50}+H]^+$, calcd. $[C_{82}H_{50}+H]$ 1035.3946; found 1035.4275. The solubility of **1c** in common deuterated solvents is insufficient to acquire ^1H or $^{13}C\{^1H\}$ NMR spectra. Samples for MAD transfer[9] and STM characterization were further purified by repetitive sonication and filtration from CH_2Cl_2.

10,10'''''-diphenyl-9,9':10',9'':10'',9''':10''',9'''':10'''',9'''''-sexianthracene (**1d**): An oven dried 100 mL Schlenk flask was charged with **7** (0.250 g, 0.491 mmol) in anhydrous Et_2O (25 mL) under N_2. The yellow suspension was cooled to –78 °C and *n*-BuLi (0.30 mL, 0.74 mmol, 2.5 M in hexanes) was added dropwise. The suspension was stirred for 2 h at –78 °C and added via cannula to a suspension of bianthrone (0.092 g, 0.24 mmol) in anhydrous Et_2O (10 mL) at 0 °C. The reaction mixture was stirred at 0 °C for 1 h then warmed to 24 °C for an additional 18 h. The suspension was quenched with AcOH (3 mL), and the precipitate was filtered and washed with EtOH to afford the dihydroxy intermediate (0.182 g, 0.146 mmol, 30%) as a yellow solid. An oven dried 5 mL sealable tube was charged with the dihydroxy intermediate (0.020 g, 0.016 mmol), $SnCl_2$ (0.040 g, 0.21 mmol), dry toluene (0.5 mL), and 1 drop of conc. H_2SO_4 under N_2. The tube was sealed and stirred at 80 °C for 16 h. The suspension was cooled to 24 °C and the solid was filtered, washed with Et_2O, H_2O, acetone, and CH_2Cl_2 to afford **1d** (1.0 mg, 0.83 μmmol, 5%) as a yellow solid. HRMS (MALDI-TOF) *m/z*: $[C_{96}H_{58}+H]^+$, calcd. $[C_{96}H_{58}+H]$ 1211.4572; found 1211.1843. The solubility of **1d** in common deuterated solvents is insufficient to acquire ^1H or $^{13}C\{^1H\}$ NMR spectra. Samples for MAD transfer[9] and STM characterization were further purified by repetitive sonication and filtration from CH_2Cl_2.

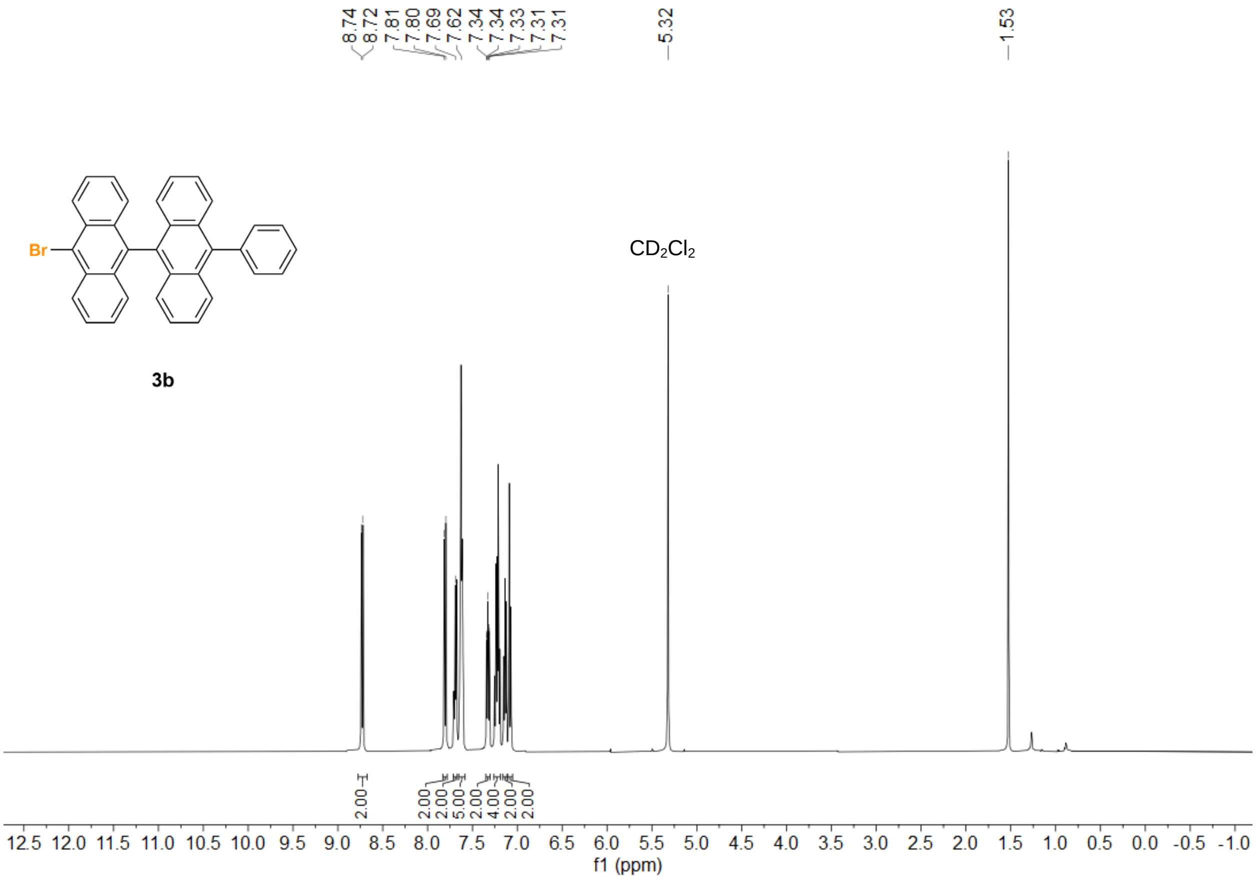

Figure S2. ^{1}H NMR (600 MHz, CD_2Cl_2) of **3b** at 24 °C.

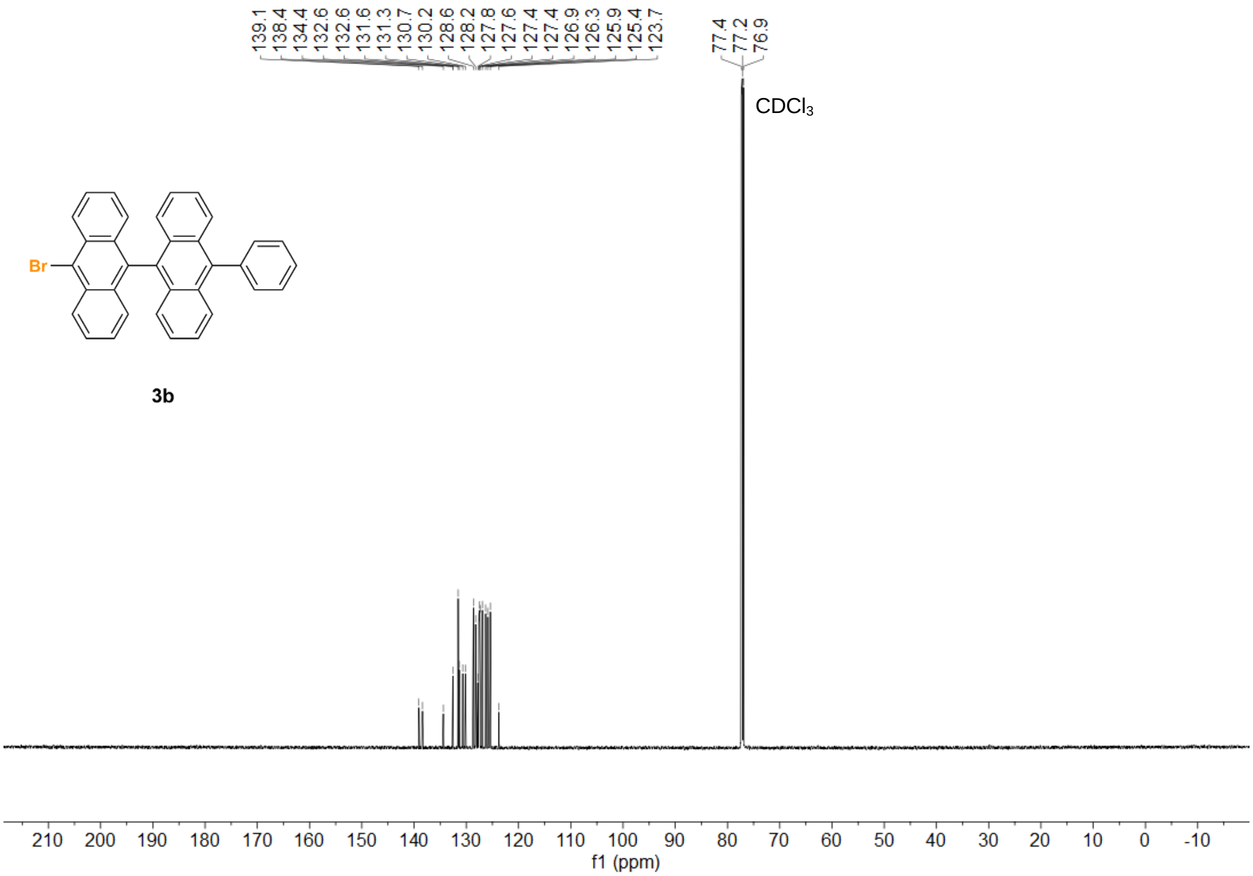

Figure S3. ^{13}C {^{1}H} NMR (151 MHz, $CDCl_3$) of **3b** at 24 °C.

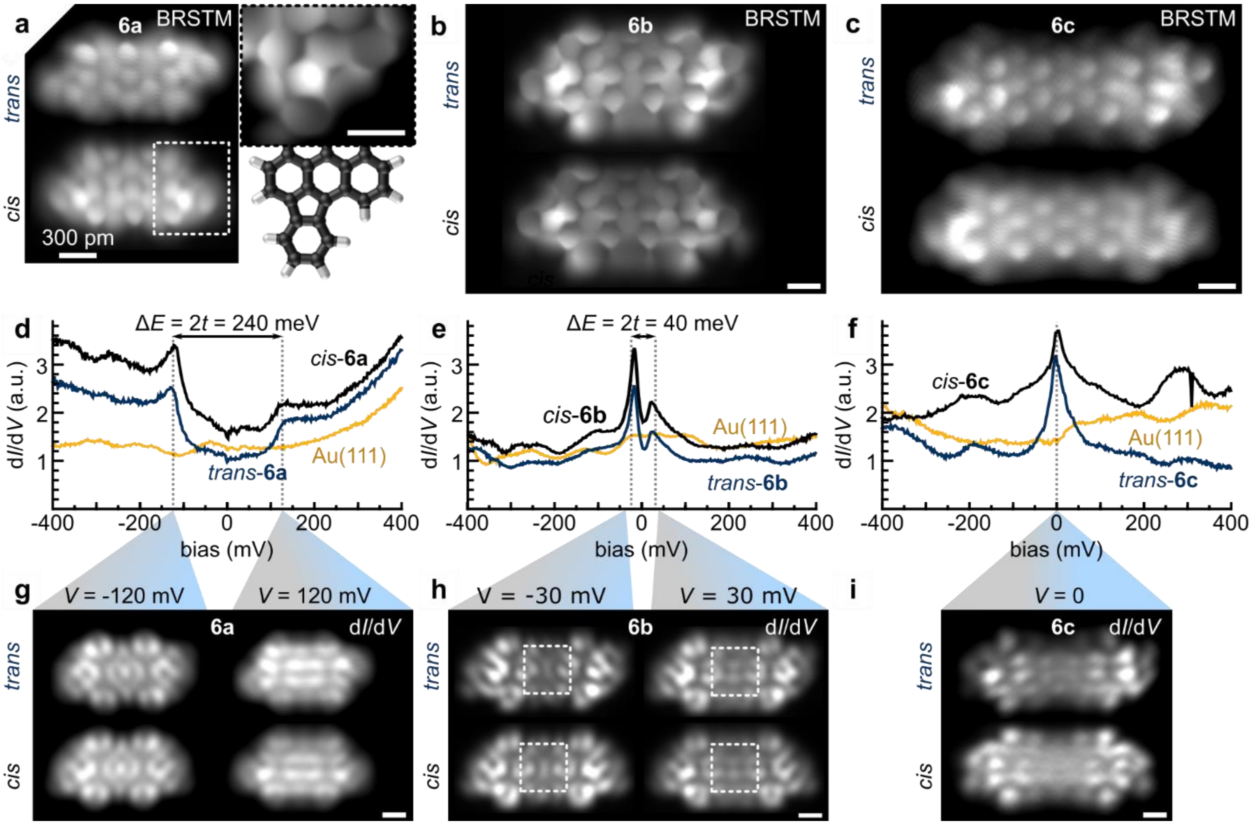

Figure S4. Experimental results on the *cis* and *trans* isomers of oligoanthenes. (a) BRSTM scans ($V = 0$, $V_{ac} = 30$ mV) of *cis*-**6a** and *trans*-**6a** (left), and a closeup BRSTM scan ($V = 350$mV, $V_{ac} = 50$mV) on the fluoranthene end (right). (b) BRSTM scans ($V = 350$ mV, $V_{ac} = 50$ mV) of *cis*-**6b** and *trans*-**6b**. (c) BRSTM scans ($V = -300$ mV, $V_{ac} = 100$ mV) of *cis*-**6c** and *trans*-**6c**. (d) STS dI/dV spectra acquired on *cis*-**6a** (black) and *trans*-**6a** (blue). (e) STS dI/dV spectra acquired on *cis*-**6b** (black) and *trans*-**6b** (blue). (f) STS dI/dV spectra acquired on *cis*-**6c** (black) and *trans*-**6c** (blue). (g) dI/dV maps ($V = –120$ mV (left), $V = 120$ mV (right), $V_{ac} = 20$ mV) of *cis*-**6a** and *trans*-**6a**. (h) dI/dV maps ($V = –30$ mV (left), $V = 30$ mV (right), $V_{ac} = 4$ mV) of *cis*-**6b** and *trans*-**6b**. (i) dI/dV maps ($V = 0$, $V_{ac} = 20$ mV) of *cis*-**6c** and *trans*-**6c**. All scale bars are 300 pm.

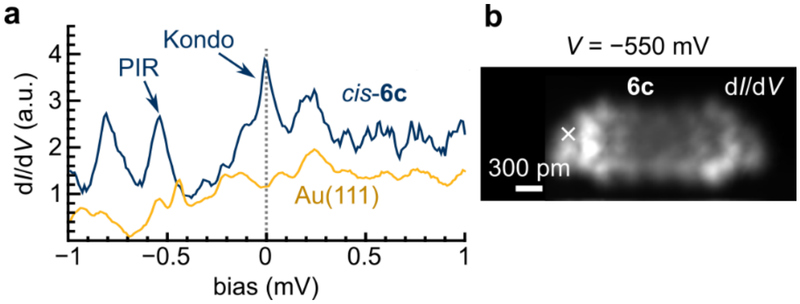

Figure S5. Identification of the positive ion resonance in **6c**. (a) Wide bias dI/dV spectrum of *cis*-**6c**, taken at the marked location in b. (b) dI/dV map ($V = –550$ mV, $V_{ac} = 50$ mV) of *cis*-**6c**.

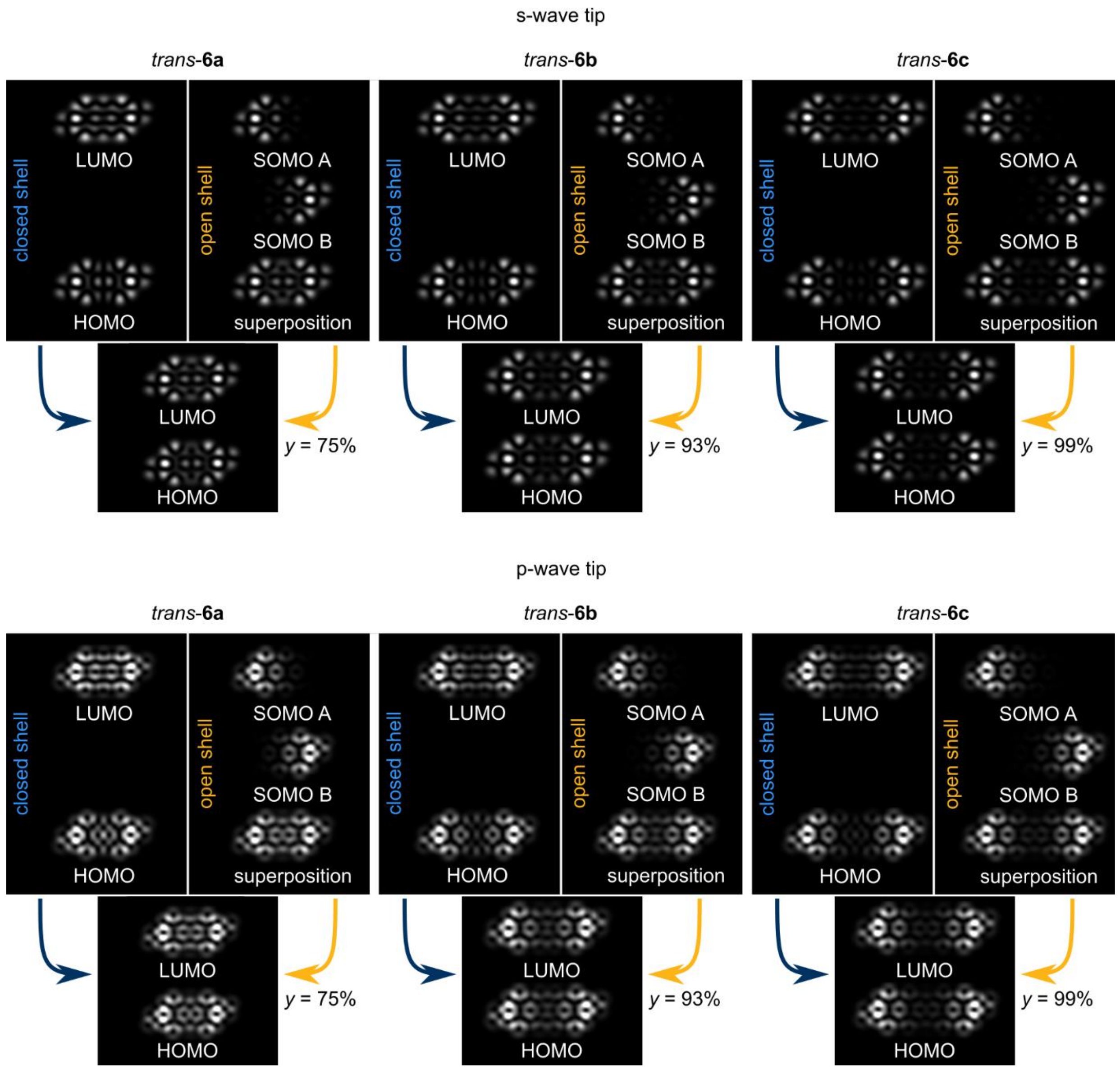

Figure S6. Simulated d*I*/d*V* maps for frontier states with s-wave (top) and p-wave (bottom) tips for **6a**, **6b**, and **6c**.

Supplementary References

(1) Friedrich, N.; Brandimarte, P.; Li, J.; Saito, S.; Yamaguchi, S.; Pozo, I.; Peña, D.; Frederiksen, T.; Garcia-Lekue, A.; Sánchez-Portal, D.; Pascual, J. I. Magnetism of Topological Boundary States Induced by Boron Substitution in Graphene Nanoribbons. *Phys. Rev. Lett.* **2020**, *125*, 146801.

(2) Cao, T.; Zhao, F.; Louie, S. G. Topological Phases in Graphene Nanoribbons: Junction States, Spin Centers, and Quantum Spin Chains. *Phys. Rev. Lett.* **2017**, *119*, 1–5.

(3) Jiang, J.; Louie, S. G. Topology Classification Using Chiral Symmetry and Spin Correlations in Graphene Nanoribbons. *Nano Letters* **2021**, *21*, 197–202.

(4) Gröning, O.; Wang, S.; Yao, X.; Pignedoli, C. A.; Borin Barin, G.; Daniels, C.; Cupo, A.; Meunier, V.; Feng, X.; Narita, A.; Müllen, K.; Ruffieux, P.; Fasel, R. Engineering of Robust Topological Quantum Phases in Graphene Nanoribbons. *Nature* **2018**, *560*, 209.

(5) Sun, Q.; Yan, Y.; Yao, X.; Müllen, K.; Narita, A.; Fasel, R.; Ruffieux, P. Evolution of the Topological Energy Band in Graphene Nanoribbons. *The Journal of Physical Chemistry Letters* **2021**, *12*, 8679–8684.

(6) Golor, M.; Koop, C.; Lang, T. C.; Wessel, S.; Schmidt, M. J.; Magnetic Correlations in Short and Narrow Graphene Armchair Nanoribbons. *Phys. Rev. Lett.* **2013**, *111*, 085504

(7) Ortiz, R.; García-Martínez, N. A.; Lado, J. L.; Fernández-Rossier, J.; Electrical spin manipulation in graphene nanostructures. *Phys. Rev. B* **2018**, *97*, 195425

(8) Asadirad, A. M.; Erno, Z.; Branda, N. R.; Photothermal Release of Singlet Oxygen from Gold Nanoparticles. *Chem. Commun.* **2013**, *49*, 5639–5641.

(9) McCurdy, R. D.; Jacobse, P. H.; Piskun, I.; Veber, G. C.; Rizzo, D. J.; Zuzak, R.; Mutlu, Z.; Bokor, J.; Crommie, M. F.; Fischer, F. R. Synergetic Bottom-Up Synthesis of Graphene Nanoribbons by Matrix-Assisted Direct Transfer. *J. Am. Chem. Soc.* **2021**, *143*, 4174–4178.
